# Effect of Connector Cross-Sectional Geometry on Fracture Resistance of Three-Unit Monolithic Zirconia Fixed Dental Prostheses Under Vertical and Oblique Loading: An In Vitro Study

**DOI:** 10.7759/cureus.113343

**Published:** 2026-07-25

**Authors:** Shazana Qazi, Shazia Kosar, Schubert Anto R, Mansi Manohar Gadhe, Adiba Ihsan, Bhushan G Wankhade

**Affiliations:** 1 Department of Prosthodontics and Crown and Bridge, Government Dental College and Hospital, Srinagar, IND; 2 Department of Prosthodontics and Crown and Bridge, Vidarbha Youth Welfare Society, Amravati's Dental College and Hospital, Amravati, IND

**Keywords:** computer-aided design-computer-aided manufacturing, dental materials, fixed dental prostheses, fracture resistance, zirconia

## Abstract

Introduction: The long-term success of monolithic zirconia fixed dental prostheses depends not only on the mechanical properties of zirconia but also on the biomechanical design of the connector region. Although connector dimensions have been extensively investigated, limited evidence is available regarding the influence of connector cross-sectional geometry on fracture resistance in different loading directions. The aim of this study was to evaluate the influence of round, triangular, and square connector cross-sectional geometries on the fracture resistance of three-unit monolithic zirconia fixed dental prostheses under vertical and oblique loading conditions.

Materials and methods: This in vitro study included 48 three-unit monolithic zirconia fixed dental prostheses fabricated using a standardized computer-aided design and computer-aided manufacturing workflow. The specimens were equally distributed into three connector design groups (round, triangular, and square), each further subdivided according to the vertical and oblique loading conditions (n = 8). The connector cross-sectional area was standardized at 9 mm² in all specimens, with the connector geometry serving as the only experimental variable. Following cementation onto the standardized resin dies, fracture resistance testing was performed using a universal testing machine at a crosshead speed of 1 mm/min until catastrophic failure. Data were analyzed using one-way analysis of variance followed by Tukey's post-hoc test, with statistical significance set at p < 0.05.

Results: The round connector demonstrated the highest mean fracture resistance under both vertical (1451.50 ± 132.84 N) and oblique (1151.25 ± 166.70 N) loading conditions. One-way analysis of variance revealed statistically significant differences among the connector designs under vertical (F = 16.50, p < 0.001) and oblique loading (F = 3.82, p = 0.038). Post-hoc analysis showed that the round connector exhibited significantly greater fracture resistance than both the triangular and square connectors under vertical loading, whereas under oblique loading, a significant difference was observed only between the round and square connector designs.

Conclusion: The connector cross-sectional geometry significantly affected the fracture resistance of monolithic zirconia fixed dental prostheses. The round connector demonstrated superior mechanical performance under both loading conditions, suggesting that rounded connector configurations may improve the structural reliability and durability of posterior zirconia fixed dental prostheses.

## Introduction

Replacement of missing teeth with fixed dental prostheses (FDPs) remains one of the most predictable treatment modalities for restoring function, esthetics, phonetics, and occlusal stability. Among the various restorative materials available, monolithic zirconia has become increasingly popular because of its excellent mechanical strength, superior fracture toughness, favorable biocompatibility, and improved optical characteristics [1,2]. Unlike other dental ceramics, zirconia exhibits transformation toughening, whereby stress-induced tetragonal-to-monoclinic phase transformation limits crack propagation, making stress distribution at the connector region a critical determinant of fracture resistance. Advances in computer-aided design and computer-aided manufacturing (CAD/CAM) technologies have further enhanced the precision and reproducibility of zirconia restorations, making them a preferred option for posterior fixed prostheses where high masticatory forces are encountered [3]. Despite these advances, mechanical failure remains a major concern, particularly in multi-unit restorations subjected to repeated functional loading.

The connector region represents the most critical biomechanical component of a three-unit FDP, serving as the structural link between the pontic and retainers [4]. During mastication, occlusal forces generate complex compressive, tensile, and shear stresses, with the highest tensile stresses concentrated at the gingival embrasure of the connector, which represents the primary site of crack initiation in zirconia fixed dental prostheses [4,5]. Unlike metallic restorations, zirconia is a brittle ceramic material that possesses excellent compressive strength, but comparatively limited resistance to tensile stress. Consequently, crack initiation and catastrophic fractures frequently originate within the connector area, making its design a major determinant of prosthesis longevity [6]. Both connector dimensions and geometric configuration influence the stress distribution and resistance to fracture under functional loading.

Although previous investigations have established minimum connector dimensions for posterior zirconia FDPs, the influence of connector cross-sectional geometry remains poorly understood [4,7]. Existing studies have reported conflicting findings regarding the mechanical performance of round, triangular, and square connector designs, and many investigations have focused primarily on vertical loading, despite the multidirectional nature of intraoral occlusal forces [7,8]. Oblique loading better simulates clinical masticatory conditions and may produce substantially different stress patterns and fracture behaviors. Therefore, evaluating connector geometry under both vertical and oblique loading conditions is essential to generate clinically relevant evidence that can guide prosthesis design [9].

Against this background, the present in vitro study was designed to compare the fracture resistance of three-unit monolithic zirconia fixed dental prostheses fabricated with round, triangular, and square connector geometries, while maintaining an identical connector cross-sectional area. This study aimed to evaluate the effect of connector cross-sectional geometry on the fracture resistance of posterior three-unit monolithic zirconia fixed dental prostheses under vertical and oblique loading conditions and to identify the connector design with the most favorable biomechanical performance.

## Materials and methods

Study design and setting

This in vitro study was conducted at the Department of Prosthodontics and Crown and Bridge, Government Dental College and Hospital, Srinagar, India, between August 2024 and December 2024. All experimental procedures were performed under standardized laboratory conditions following a predetermined protocol to minimize operator- and procedure-related variability.

Sample size calculation

The sample size was determined a priori using the G*Power software version 3.1 (Heinrich Heine University Düsseldorf, Düsseldorf, Germany). The calculation was based on a one-way fixed-effects omnibus analysis of variance (ANOVA) using an effect size (f) of 0.60 obtained from a previous in vitro study evaluating connector designs in monolithic zirconia fixed dental prostheses [10]. Assuming a significance level (α) of 0.05, statistical power of 80%, and six study groups, the minimum required sample size was calculated to be 48 specimens. Accordingly, eight specimens were allocated to each subgroup, providing an actual statistical power of 85.8%, which was considered adequate to detect statistically significant differences among connector designs.

Specimen preparation and calibration

A mandibular typodont model, simulating the absence of the mandibular first molar, was selected for this study. The mandibular second premolars and second molars were prepared by a single experienced operator to receive a three-unit fixed dental prosthesis. Standardized tooth preparation parameters were maintained, including a total occlusal convergence of 6°, a 0.5-mm deep chamfer finish line, an axial wall height of 4-5 mm, a 1.5 mm occlusal reduction, and rounded internal line angles. Prior to specimen fabrication, a pilot standardization procedure was performed to ensure the reproducibility of the preparation design and digital workflow. The prepared typodont was scanned twice at a two-week interval, and all digital measurements of connector dimensions were repeated using the measurement tools incorporated in the Exocad DentalCAD software (Exocad GmbH, Darmstadt, Germany). Intra-examiner reliability was assessed using the intraclass correlation coefficient (ICC), and an ICC value greater than 0.90 was considered indicative of excellent measurement reliability before commencement of the definitive experiment.

Digital design and fabrication of specimens

The prepared typodonts were digitized using a UP400 high-speed dental laboratory scanner (Shenzhen UP3D Tech Co., Ltd., Shenzhen, Guangdong, China), and stereolithography (STL) data were imported into Exocad DentalCAD software (Exocad GmbH, Darmstadt, Germany). A standardized three-unit monolithic zirconia fixed dental prosthesis replacing the mandibular first molar was digitally designed using a modified ridge-lap pontic. The connector cross-sectional area was maintained at 9 mm² for all specimens, whereas only the connector geometry was altered. Three connector configurations were evaluated: round, triangular, and square configurations. The triangular connector was designed with its base oriented toward the gingival aspect, whereas the square and round connectors were designed with equivalent cross-sectional areas to isolate the effect of the geometry. The master die was fabricated using a high-strength three-dimensional printable resin, and all zirconia restorations were milled from IPS e.max ZirCAD LT zirconia blanks (Ivoclar AG, Schaan, Liechtenstein), a 3 mol% yttria-stabilized tetragonal zirconia polycrystal (3Y-TZP), using a computer-aided design and computer-aided manufacturing (CAD/CAM) milling system. The restorations were subsequently sintered according to the manufacturer's recommendations before finishing and polishing. The completed restorations were subsequently finished and polished using standardized procedures. All connector designs were generated using standardized CAD parameters while maintaining an identical connector cross-sectional area across all groups. Although the internal embrasure curvature was determined by the CAD software based on the selected connector geometry, the same design protocol was applied consistently to all specimens to minimize geometric variability.

Cementation procedure

The intaglio surfaces of all zirconia restorations were treated with 10-methacryloyloxydecyl dihydrogen phosphate (MDP)-containing zirconia primer, after which the restorations were cemented onto their corresponding resin dies using a Coltene dual-cure resin cement (Coltene Holding AG, Altstätten, Switzerland). A standardized seating pressure was maintained during cementation to ensure a uniform film thickness. Following cementation, all specimens were stored in distilled water at 37°C for 24 h to allow complete polymerization of the luting cement and simulate intraoral conditions before mechanical testing.

Fracture resistance testing

The cemented specimens were randomly allocated to either the vertical or oblique loading groups. Mechanical testing was conducted using a calibrated Universal Testing Machine. Each specimen was positioned on a custom testing jig, and a load was applied to the central fossa of the pontic using a 5-mm diameter stainless steel spherical indenter at a crosshead speed of 1 mm/min until catastrophic fracture occurred. For the vertical loading group, the load was applied perpendicular to the occlusal plane, whereas for the oblique loading group, the load was applied at an angle of 45° to the long axis of the prosthesis to simulate non-axial occlusal loading. The maximum fracture load at failure was recorded in Newtons (N) for each specimen.

Statistical analysis

The collected data were analyzed using the Statistical Package for the Social Sciences (SPSS) version 25.0 (IBM Corp., Armonk, NY, USA). Data normality was assessed using the Shapiro-Wilk test. Continuous variables were summarized as mean, standard deviation (SD), and 95% confidence intervals (CI). Separate one-way analyses of variance (ANOVA) were performed to compare the fracture resistance among the connector designs under vertical and oblique loading conditions. Whenever the overall ANOVA demonstrated statistically significant differences, Tukey's honestly significant difference (HSD) post-hoc test was used for pairwise comparisons. The effect size was calculated using eta squared (η²) to quantify the magnitude of group differences. A two-tailed p < 0.05 was considered statistically significant for all analyses.

## Results

A total of 48 three-unit monolithic zirconia fixed dental prostheses were included in the study, with eight specimens allocated to each experimental subgroup. The descriptive statistics for the fracture strength under vertical and oblique loading conditions are presented in Table 1. Under vertical loading, the round connector design demonstrated the highest mean fracture strength (1451.50 ± 132.84 N), followed by the triangular (1128.63 ± 126.75 N) and square (1091.50 ± 152.64 N) connector designs. A similar trend was observed under oblique loading, where the round connector exhibited the greatest mean fracture strength (1151.25 ± 166.70 N), while the triangular and square connectors showed comparatively lower fracture resistance (Table 1).

**Table 1:** Descriptive statistics of fracture strength according to connector design under vertical and oblique loading. Data are presented as mean ± standard deviation (SD) and 95% confidence interval (CI), n indicates the number of specimens in each subgroup, fracture strength is expressed in Newtons (N).

Parameter	Connector design	Frequency	95% Confidence interval for mean	Mean ± SD
Vertical load	Round	8	1340.44-1562.56	1451.50 ± 132.84
	Triangular	8	1022.66-1234.59	1128.63 ± 126.75
	Square	8	963.89-1219.11	1091.50 ± 152.64
Oblique load	Round	8	1011.88-1290.62	1151.25 ± 166.70
	Triangular	8	886.38-1140.12	1013.25 ± 151.76
	Square	8	908.43-1031.57	970.00 ± 73.64

Intergroup comparisons using one-way analysis of variance demonstrated statistically significant differences in fracture strength among the three connector designs under both loading conditions (Table 2). Under vertical loading, the connector geometry accounted for a large proportion of the variation in fracture resistance (F = 16.50, p < 0.001, η² = 0.61). Under oblique loading, a statistically significant difference was also observed (F = 3.82, p = 0.038, η² = 0.27), indicating a moderate effect of the connector design on the fracture resistance.

**Table 2:** Comparison of fracture strength among connector designs under vertical and oblique loading using one-way analysis of variance. Data were analyzed using one-way analysis of variance (ANOVA), η² represents the effect size (eta squared), df: degrees of freedom, *p < 0.05 was considered statistically significant.

Parameter	Interaction	Sum of squares	df	Mean square	F value	p-value	Effect size (η^2^)
Vertical load	Group	627270.8	2	313635.38	16.50	<0.001*	0.61
	Residual	399085.9	21	19004.09			
Oblique load	Group	143376.3	2	71688.17	3.82	0.038*	0.27
	Residual	393699.0	21	18747.57			

Post-hoc pairwise comparisons using Tukey's test revealed that under vertical loading, the round connector design exhibited significantly greater fracture strength than both the triangular and square connector designs (p < 0.001 for both comparisons), whereas no significant difference was observed between the triangular and square groups. Under oblique loading, the round connector demonstrated significantly greater fracture resistance than the square connector (p = 0.045), whereas the remaining pairwise comparisons were not statistically significant (Table 3).

**Table 3:** Pairwise comparison of fracture strength among connector designs using Tukey's post-hoc test. Pairwise comparisons were performed using Tukey's honestly significant difference (HSD) post-hoc test following one-way analysis of variance, data are presented as mean differences with 95% confidence intervals (CI), t: t statistic, CI: confidence interval, *p < 0.05 was considered statistically significant.

Parameter	Pairwise group	Mean difference	t-value	p-value	95% CI lower limit	95% CI upper limit
Vertical load	Round-Triangular	322.88	4.68	<0.001*	143.57	502.18
	Round-Square	360.00	5.22	<0.001*	180.70	539.30
	Triangular-Square	37.13	0.54	1.000	-142.18	216.43
Oblique load	Round-Triangular	138.00	2.02	0.170	-40.09	316.09
	Round-Square	181.25	2.65	0.045*	3.16	359.34
	Triangular-Square	43.25	0.63	1.000	-134.84	221.34

## Discussion

The present in vitro study evaluated the influence of three connector cross-sectional geometries (round, triangular, and square) on the fracture resistance of three-unit monolithic zirconia fixed dental prostheses under vertical and oblique loading conditions, while maintaining a constant connector cross-sectional area of 9 mm². The findings demonstrated that the connector geometry significantly influenced the fracture resistance under both loading conditions. The round connectors consistently exhibited the highest fracture resistance, whereas the triangular and square connectors exhibited comparatively lower values. These findings support the concept that, even when the connector area is standardized, geometric configuration plays an important role in the stress distribution and mechanical performance of zirconia restorations.

The triangular connector in the present study was designed with its base oriented toward the gingival aspect to provide a broader gingival embrasure contour while maintaining a constant connector cross-sectional area. This configuration was selected to avoid a sharp gingival apex, which may increase tensile stress concentration and facilitate crack initiation. Clinically, a broader gingival connector contour is also more consistent with conventional prosthesis design, as it permits smoother stress distribution while maintaining an appropriate embrasure form that facilitates oral hygiene and respects the biological width.

Under vertical loading, the round connector demonstrated a significantly greater fracture resistance than both the triangular and square connector designs. This finding can be explained by the round connector's smoother curvature, which promotes a more uniform distribution of compressive and tensile stresses throughout the connector region. Rounded geometries minimize abrupt changes in contour and reduce stress concentration at the gingival embrasure, which is recognized as the most vulnerable region for crack initiation in all-ceramic fixed dental prostheses [11]. In contrast, triangular and square connectors contain relatively sharper transitions that increase the localized tensile stresses and facilitate crack propagation under increasing loads. Consequently, round connectors are better able to withstand higher occlusal forces before catastrophic failure occurs.

The findings of the present study differ from those reported by Muhsin et al. [12], who performed a finite element analysis of implant-supported metal fixed partial dentures and observed no significant differences in stress distribution or deformation between the round and square connector designs. This discrepancy may be attributed to methodological differences. Their investigation employed a virtual finite element model of a cobalt-chromium metal framework, whereas the present study evaluated the fracture resistance of monolithic zirconia fixed dental prostheses under destructive mechanical loading. Furthermore, the biomechanical behavior of ductile metallic frameworks differs substantially from that of brittle ceramic materials, such as zirconia, in which fracture is predominantly governed by the tensile stress concentration at the connector region. Differences in supporting structures, material properties, loading protocols, and outcome measures may therefore account for these contrasting findings.

The superior mechanical performance of the round connector is consistent with the findings of Oh and Anusavice [11], who reported that rounded connector configurations significantly improve fracture resistance by reducing the stress concentration within ceramic fixed partial dentures. These findings are supported by the finite element analysis conducted by Alberto et al. [13], who demonstrated that increasing the radius of curvature at the gingival embrasure significantly reduced the peak tensile stresses and promoted a more homogeneous stress distribution within all-ceramic fixed dental prostheses. Beuer et al. [14] also observed that rounded connectors fabricated using CAD/CAM technology exhibited improved biomechanical behavior and reduced susceptibility to fractures. These studies collectively support the present findings that connector geometry, independent of connector size, contributes substantially to the longevity of zirconia fixed dental prostheses.

Although the triangular connector demonstrated a greater mean fracture resistance than the square connector under vertical loading, the difference between the two designs was not statistically significant. This observation suggests that once sharp geometric transitions are introduced, differences in the stress distribution become less pronounced, despite variations in the connector shape. Because the connector cross-sectional area remained constant in the present study, the observed differences were primarily attributable to the connector morphology rather than the material volume. This finding further emphasizes that the connector geometry should be optimized in conjunction with adequate connector dimensions during the prosthesis design [15].

Under oblique loading, the fracture resistance was reduced in all three connector designs compared with vertical loading. This finding reflects the multidirectional nature of occlusal loading, in which oblique forces generate greater bending moments and tensile stresses than do purely axial loading. Ceramic materials exhibit excellent compressive strength but comparatively lower tensile strength; therefore, non-axial forces increase the likelihood of crack initiation and propagation within the connector region [16]. Nevertheless, the round connector continued to demonstrate the highest fracture resistance, and a statistically significant difference was observed between the round and square connector designs. The absence of statistically significant differences between the round and triangular connectors, or between the triangular and square connectors, may be attributed to the greater variability associated with oblique loading, which is reflected by the wider dispersion of fracture values.

The reduction in the fracture resistance under oblique loading agrees with the observations of Ahmed et al. [8], who reported that oblique forces produce lower fracture loads than vertical forces, regardless of the connector design. The present findings differ from those of Khorshed et al. [17], who reported significantly higher fracture resistance for oval connectors than for circular connectors in long-span implant-supported monolithic zirconia fixed partial dentures. This discrepancy may be attributed to differences in prosthesis design, as their study evaluated four-unit implant-supported restorations with varying connector sizes, whereas the present study investigated three-unit tooth-supported prostheses with a standardized connector area. These methodological differences may have influenced the observed biomechanical behavior.

The large effect size observed under vertical loading and the moderate effect size under oblique loading indicate that connector geometry has a greater influence on fracture resistance during axial loading than during oblique loading. Under oblique loading, additional bending and shear components likely contribute to the fracture behavior irrespective of the connector shape, thereby reducing the relative contribution of the geometry alone. Nevertheless, the persistence of significant differences under both loading conditions suggests that the connector configuration remains an important design parameter in posterior zirconia restorations.

From a clinical perspective, these findings suggest that rounded connector designs are preferred whenever anatomical and prosthetic conditions permit. Improved fracture resistance associated with rounded connectors may contribute to the enhanced longevity of posterior monolithic zirconia fixed dental prostheses, particularly in patients with high occlusal loads or parafunctional habits. As CAD/CAM technology enables precise customization of connector geometry, incorporating rounded connector designs into routine digital workflows may improve the mechanical reliability of zirconia restorations without increasing connector dimensions or compromising esthetics.

Despite these encouraging findings, this study has some limitations. It was conducted under controlled laboratory conditions that could not completely reproduce the complex oral environment. Factors such as thermal cycling, cyclic fatigue loading, moisture degradation, and the associated low-temperature degradation (LTD) of zirconia, aging, occlusal wear, and parafunctional activities were not simulated and may influence long-term clinical performance. In addition, periodontal ligament simulation was not incorporated, as the specimens were rigidly mounted during mechanical testing. Consequently, the physiological mobility of natural teeth and the resulting stress distribution within the connector region may not have been fully replicated. Only a single connector cross-sectional area (9 mm²), one zirconia material, and a three-unit posterior fixed dental prosthesis configuration were evaluated. Furthermore, although a 9 mm² connector area has been widely used in experimental studies, contemporary zirconia systems may recommend larger minimum connector dimensions for posterior multi-unit fixed dental prostheses. Therefore, the results should be interpreted with caution when extrapolating them to different connector dimensions, zirconia generations, longer-span prostheses, or implant-supported restorations. Future studies incorporating artificial aging protocols, periodontal ligament simulation, fatigue testing, finite element analysis, and long-term clinical evaluation are warranted to further validate the biomechanical advantages of different connector geometries.

## Conclusions

The cross-sectional geometry of the connector significantly influenced the fracture resistance of three-unit monolithic zirconia fixed dental prostheses. Among the evaluated designs, the round connector demonstrated the highest mean fracture resistance under both vertical and oblique loading conditions. Under vertical loading, the round connector exhibited significantly greater fracture resistance than the triangular and square connectors, whereas under oblique loading, it was significantly stronger than the square connector but showed no statistically significant difference compared with the triangular connector. These findings highlight the importance of optimizing connector geometry during CAD/CAM prosthesis design to improve the structural integrity and potential clinical longevity of posterior monolithic zirconia fixed dental prostheses.
